# The impact of Taiwan’s implementation of a nationwide harm reduction program in 2006 on the use of various illicit drugs: trend analysis of first-time offenders from 2001 to 2017

**DOI:** 10.1186/s12954-021-00566-5

**Published:** 2021-11-19

**Authors:** Wei J. Chen, Chi-Ya Chen, Shang-Chi Wu, Kevin Chien-Chang Wu, Susyan Jou, Yu-Chi Tung, Tzu-Pin Lu

**Affiliations:** 1 Institute of Epidemiology and Preventive Medicine, College of Public Health, National Taiwan University, 17 Xu-Zhou Road, Taipei, 100 Taiwan; 2 Department of Public Health, College of Public Health, National Taiwan University, Taipei, Taiwan; 3 Department of Psychiatry, College of Medicine and National Taiwan University Hospital, National Taiwan University, Taipei, Taiwan; 4 Center for Neuropsychiatric Research, National Health Research Institutes, Zhunan Town, Miaoli County Taiwan; 5 Graduate Institute of Medical Education and Bioethics, College of Medicine, National Taiwan University, Taipei, Taiwan; 6 Graduate School of Criminology, National Taipei University, New Taipei, Taiwan; 7 Institute of Health Policy and Management, College of Public Health, National Taiwan University, Taipei, Taiwan

**Keywords:** Injection drug use, Harm reduction, Segmented regression analysis, Time trend, Heroin, Intervention, Diffusion effect

## Abstract

**Background:**

After implementing a nationwide harm reduction program in 2006, a dramatic decline in the incidence of human immunodeficiency virus (HIV) infection among people with injection drug use (IDU) was observed in Taiwan. The harm reduction program might have sent out the message discouraging the choice of IDU among illicit drug users in early stage. Based on the yearly first-time offense rates from 2001 to 2017, this study aimed to examine (1) whether the nationwide implementation of the harm reduction program in 2006 led to changes in first-time offenders’ use of heroin; (2) whether the intervention had a similar effect on the use of other illicit drugs; and (3) whether the effect of the intervention was limited to the first-time offenders of young age groups.

**Methods:**

Yearly first-time illicit-drug offense rates from 2001 to 2017 in Taiwan were derived from two national databases for drug arrests that were verified using urine tests: the Criminal Record Processing System on Schedule I/II Drugs and the Administrative Penalty System for Schedule III/IV Substances. A hierarchy of mutually exclusive categories of drug uses was defined by the drug with the highest schedule level among those tested positive in an arrest. Segmented regression analyses of interrupted time series were used to test for the impact of the 2006 intervention.

**Results:**

There was a decrease of 22.37 per 100,000 in the rate for heroin but no detectable level changes in that for methamphetamine or ecstasy after the 2006 intervention in Taiwan. There were baseline decreasing trends in the first-time offense rate from 2001 to 2017 for heroin and ecstasy and an increasing trend for methamphetamine, with the slopes not altered by the 2006 intervention. The postintervention decrease in the first-time offense rate for heroin was detectable among offenders less than 40 years old.

**Conclusions:**

Our results indicate a diffusion effect of the 2006 intervention on decreasing heroin use among young offenders and have policy implications for better prevention and treatment for different age groups.

**Supplementary Information:**

The online version contains supplementary material available at 10.1186/s12954-021-00566-5.

## Background

Illicit drug use has been an important contributor to the global disease burden. An estimated 5.5% of the global population aged 15–64 years old in 2017 had used illicit drugs in the previous year, which was 30% more than that in 2009 [1]. Globally, 1.3% of all disability-adjusted life-years were attributable to drug use as a risk factor in 2016 [2]. Among them, injection drug use (IDU) has been of intense concern because of its increased risk of overdose-related death and contracting infections such as human immunodeficiency virus (HIV) and hepatitis C virus [3]. Until the early 2010s, IDU remained a challenging issue in regions such as Asia, where the incidence of HIV infection continued to increase [4]. Despite empirical evidence showing that harm reduction programs are effective in decreasing IDU-related harm, their adoption was slow in Asia owing to the concern over spillover effects, such as the diversion of the opioid agonist medications [5, 6], sending a wrong or pro-drug message to the public [7], and traditional emphasis on strict enforcement of punitive national antinarcotic laws [8–10].

In the past three decades, Taiwan has witnessed drastic changes in drug use, and the government has developed policy changes over time. In the 1990s, methamphetamine use surged, and it became the most common illicit drug for those incarcerated. This increase triggered the promulgation of a new law in 1998, the “Narcotics Hazard Prevention Act”, that treated an addict similar to a “diseased criminal” rather than simply a “criminal” [11]. Then, the classification of ecstasy was elevated to Schedule II (i.e., its users are subject to criminal prosecution) in 1999 because of its increased popularity among young people [12].

Taiwan’s illicit drug control policy made another significant change that was triggered by a major HIV epidemic that emerged among people with IDU in 2004, accounting for 72% of newly reported HIV cases in that year [13]. Following a pilot carried out in four major sites in 2005 [14], a three-pronged harm reduction program, including the expansion of extant education and screening, a needle–syringe program (NSP), and the opioid substitution therapy (OST) [13, 15], was implemented nationwide, i.e., in every city and county, in 2006. With a fast scaling-up in NSP sites that was rated as a successful model in a systemic review [16], a dramatic decline in the incidence of HIV infection among people with IDU was observed [17]. Remarkably, the percentage of all newly reported HIV cases attributable to IDU fell from 72% in 2004 to 2% in 2018 [18]. Under this circumstance, the harm reduction program might have sent out the message discouraging the choice of IDU among illicit drug users in early stage, resulting in a decrease in first-time offense rate of heroin.

Furthermore, legal amendments to allow deferred prosecution nationwide were enacted in 2008. Then, in 2009, another amendment to the law stipulated that any adult who used or possessed less than 20 g of ketamine, a Schedule III substance, would have to pay a fine and be forced to attend a drug seminar as their penalty; individuals would be criminally prosecuted if the weight was 20 g or more [19].

The complexity of public health interventions in the real-world settings often poses methodological challenges for their evaluations [20]. For the case of Taiwan’s implementation of a harm reduction program in 2006, it would be more realistic to adopt an interrupted time-series approach in such evaluations [21, 22]. Recently, the Taiwanese government assembled electronic databases that enroll adults who were arrested for drug offenses. These databases cover all kinds of illicit drugs that tested positive and allow researchers to create national cohorts of first-time offenders, who were presumably in relatively early stage of illicit drug use [23]. This provided an opportunity to examine the impact of the harm reduction program on first-time drug offenders’ choice of illicit drugs over time. We hypothesized that after the nationwide implementation of harm reduction, the hidden community of illicit drug users would be more aware of the risk associated with IDU and might change from using injected drugs to those that could be administered orally or in other less invasive ways. To evaluate the variations in the use of different illicit drugs, we applied a hierarchical classification of illicit drugs if a person tested positive for more than two kinds of illicit drugs. Based on the yearly first-time offense rates from 2001 to 2017, this study aimed to examine (1) whether the nationwide implementation of the harm reduction program in 2006 led to changes in first-time offenders’ use of heroin; (2) whether the intervention had a similar effect on the use of other illicit drugs; and (3) whether the effect of the intervention was limited to the first-time offenders of young age groups.

## Methods

### Study samples

We obtained study samples from two national databases of illicit drug offenses in Taiwan. The first database, known as the Criminal Record Processing System (CRPS), has electronic data from drug offenders enrolled for using Schedule I (e.g., heroin and morphine) or II (e.g., methamphetamine, marijuana, and ecstasy) substances with verification using urine tests since 2001. The second, known as the Administrative Penalty System for Schedule III/IV Substances (APS), has electronic data from drug offenders enrolled for using Schedule III (e.g., ketamine) or IV (e.g., 5-Methoxy-N,N-diisopropyltryptamine or Foxy) substances. The APS was created following the legal amendment in November 2009 stipulating that any adult who used or possessed less than 20 g of ketamine would have to pay a fine of NT$ 10,000 to 50,000 (approximately US$ 333 to 1667) and be required to attend a four- to eight-hour drug seminar as a penalty; individuals would be criminally prosecuted if the weight was 20 g or more [19]. Data were retrieved from both databases from their initiation date until the end of 2017. Data cleaning procedures were conducted by excluding those without a valid identification number and those aged 80 or over.

Both the APS and CRPS are maintained by Taiwan’s National Police Agency. Before being transferred to the Health and Welfare Data Science Center for research, all offenders’ information undergoes a deidentification process using a scrambling procedure that allows for records to be linked but without any revelation of personal identifiers. The study was approved by the Research Ethics Committee of National Taiwan University Hospital (NTUH-REC no. 201802050RINC).

### The nationwide harm reduction program

The nationwide harm reduction program implemented by the Taiwanese government in 2006 consisted of three parts [13–15]. The first part was called information, education, and communication via expansion of existing screening and education service to people with IDU, with emphasis on avoiding the reuse of drug paraphernalia and the sharing of dissolved heroin solution [24]. The second part was the NSP that distributed clean needles and syringes and collected used ones for safe disposal, as well as distributed free condoms and educational materials regarding the prevention of blood or sexually transmitted disease. In a fast scaling-up in the first year, Taiwan Centers for Disease Control established 729 NSP sites that were either pharmacy-based NSP sites or vending machines and distributed 438,081 items of needles–syringes in 2006 [13]. The third part was the OST provided in a number of public hospitals, where HIV-infected person with IDU could receive methadone treatment free of charge owing to the support by a special governmental funding for the control of HIV, while HIV seronegative ones were charged ca. US$1600 per year for the same treatment [17]. In the first year, there were 21 OST clinics with 641 cases on treatment [13]. Both the number of needles–syringes distributed in NSP sites and the number of cases on OST clinics continued to increase in subsequent years and peaked in 2008 [13].

### Classification of drug offenders

Every arrest involving an illicit drug offense was enlisted in either the CRPS or APS and was called a drug offense event in this study. However, the two databases did not have information about whether an arrestee had a previous offense record. Following our previous study [23], we adopted an operational approach toward the classification of drug offenders. First, we classified the drug arrest events by calendar year. Then, we counted the unique individuals in each year as prevalent offenders. Finally, the first year that an offender appeared in the databases was determined, and the offender was classified as a first-time offender in that particular year. Any appearance of the offender in the database after that year led the person to be classified as a repeat offender.

We adopted a hierarchy of mutually exclusive categories of drug use following the principle of a previous study [25]. Specifically, we classified drug use categories by the highest level of scheduling in all the drugs used by a person in each year: (1) heroin (Schedule I) use, regardless of use of the other drugs; (2) methamphetamine (Schedule II) use, no use of heroin; (3) ecstasy (Schedule II) use, no use of heroin or methamphetamine; (4) ketamine (Schedule III) criminal use (manufacturing or selling, or possessing ≥ 20 g since 2009), no use of heroin, methamphetamine, or ecstasy; (5) ketamine noncriminal use (use or possessing < 20 g since 2009), no use of heroin, methamphetamine, ecstasy, or ketamine criminal use; and (6) use of other drugs.

### Statistical analysis

Based on the age-specific rate of both prevalent and first-time offenders, we then calculated an age-standardized offense rate using the World Standard population (WHO 2000–2025) [26], truncated to the age range between 18 and 69 years, as the weighting for the population.

Then, we used segmented regression analysis of interrupted time series to evaluate the impact of a policy change on the yearly age-standardized first-time offense rate, in which the time period was divided into pre- and postintervention segments, and separate intercepts and slopes were estimated in each segment [27]. Briefly, we specified the following linear regression model to estimate the level and trend in the age-standardized yearly first-time offense rate before the 2006 harm reduction program and the changes in level and trend following the harm reduction program:

$$Y_{t} = {\text{b}}_{0} + {\text{b}}_{{1}} \times T_{t} + {\text{b}}_{{2}} \times Int_{t} + {\text{b}}_{{3}} \times TA_{t} + e_{t}$$

where *Y*_*t*_ is the yearly first-time offense rate in year t; *T*_*t*_ indicates time in year at time t from the start of the observed period (2001 to 2017); *Int*_*t*_ is an indicator for time t occurring before (*Int*_*t*_ = 0) or after (*Int*_*t*_ = 1) the harm reduction program, which was implemented in year 6; *TA*_*t*_ indicates time in year after intervention at time t. In this model, β_0_ estimates the baseline level of the outcome (i.e., the yearly first-time offense rate in 2001); β_1_ estimates the baseline slope in the first-time offense rate before the intervention; β_2_ estimates the level change in the first-time offense rate immediately after the intervention; and β_3_ estimates the additional change in the slope in the first-time offense rate after the intervention. Thus, the sum of β_1_ and β_3_ is the postintervention slope. All statistical analyses were conducted using SAS version 9.4 (SAS Institute Inc., Cary, NC).

## Results

### First-time offenders

**Fig. 1:**
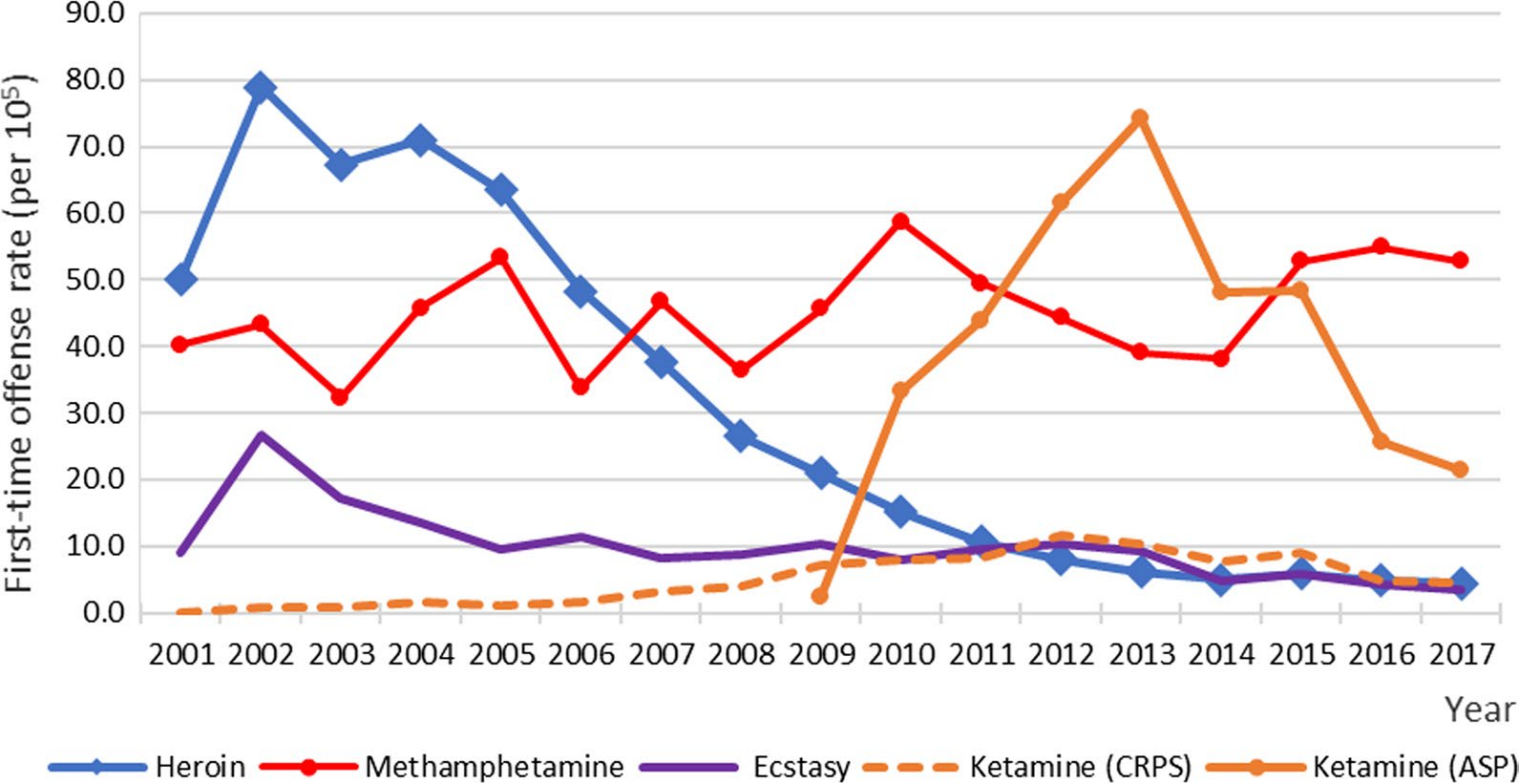
Yearly age-standardized first-time offense rate from 2001 to 2017 for five hierarchically classified kinds of illicit drugs, including heroin, methamphetamine, ecstasy, ketamine (CRPS, i.e., Criminal Record Processing System), and ketamine (ASP, i.e., Administrative Penalty System) in Taiwan from 2001 to 2017

From 2001 to 2017, a total of 889,721 drug offenses were committed by 586,650 persons (referred to as prevalent offenders hereafter), 261,085 of whom were first-time drug offenders (Additional file 1: Table S1). Male offenders constituted the majority of each year’s offenders, ranging from 81.8% (13,062 out of 15,976) in 2011 to 85.6% (12,208 out of 14,255) in 2001 (Additional file 1: Table S2). When a hierarchical classification of illicit drug offenses was applied, the distribution of different categories of illicit drugs from 2001 to 2017 is shown in Table 1. The first-time offense rates for hierarchically classified five categories of illicit drugs, including heroin, methamphetamine, ecstasy, ketamine-criminal use, and ketamine-noncriminal use, from 2001 to 2017 are shown in Fig. 1.

**Table 1:** Hierarchical classification of the first-time illicit drug offenders in Taiwan, 2001–2017

	First-time offenders		Heroin		Methamphetamine		Ecstasy		Ketamine (CRPS)		Ketamine (APS)		Others
Year	N	R (/10^5^)^a^	n	R (/10^5^)^a^	n	R (/10^5^)^a^	n	R (/10^5^)^a^	n	R (/10^5^)^a^	n	R (/10^5^)^a^	n
2001	14,255	91.39	6928	50.00	5518	40.21	1191	8.93	0	0.00	–	–	618
2002	21,423	173.49	10,992	78.90	5963	43.24	3534	26.72	98	0.74	–	–	836
2003	16,963	137.26	9422	67.32	4493	32.34	2240	17.07	124	0.93	–	–	684
2004	18,974	153.62	9977	70.94	6369	45.72	1760	13.58	194	1.50	–	–	674
2005	18,347	146.64	8984	63.40	7410	53.31	1205	9.44	140	1.09	–	–	608
2006	13,884	108.45	6936	48.31	4722	33.74	1420	11.27	219	1.70	–	–	587
2007	13,974	109.38	5429	37.65	6475	46.72	1046	8.30	389	3.10	–	–	635
2008	11,034	86.34	3857	26.50	5022	36.42	1081	8.61	482	3.93	–	–	592
2009	12,176	97.25	3053	20.93	6188	45.61	1282	10.33	855	7.12	289	2.42	509
2010	16,538	137.15	2207	15.19	7926	58.77	970	7.88	945	8.03	3975	33.36	515
2011	15,976	135.39	1561	10.63	6689	49.50	1158	9.53	948	8.14	5208	43.90	412
2012	17,201	148.53	1175	8.00	5966	44.26	1254	10.38	1339	11.69	7253	61.54	214
2013	17,663	155.31	916	6.17	5251	38.98	1156	9.39	1191	10.36	8756	74.11	393
2014	13,185	112.34	748	5.01	5110	38.11	594	4.81	887	7.76	5610	48.08	236
2015	15,486	131.06	863	5.89	6976	52.73	703	5.91	1032	9.09	5600	48.36	312
2016	12,382	104.15	724	4.82	7204	54.82	492	4.17	547	4.86	2977	25.76	438
2017	11,623	96.69	800	4.42	7001	52.83	399	3.44	485	4.41	2416	21.32	522

^a^ Age-standardized first-time offense rate using the World Standard population (WHO 2000–2025) [26], truncated to the age range between 18 and 69 years, as the weighting for the population (18–24 years: 18.24%; 25–29 years: 12.49%; 30–39 years: 23.24%; 40–49 years: 19.89%; 50–59 years: 15.62%; and 60–69 years: 10.52%)

**Table 2:** Time series modeling of age-standardized first-time offense rates for heroin and methamphetamine, from 2001 to 2017 in Taiwan

Parameters in the parsimonious model	Coefficient estimate	Standard error	t‐statistic	*P* value
First-time offense rate for heroin				
Intercept (β_0_)	75.86	4.63	16.38	< .0001
Baseline slope (β_1_)	− 3.25	0.74	− 4.42	0.001
Level change after intervention (β_2_)	− 22.37	7.91	− 2.83	0.01
β_2_/β_1_	6.88			
First-time offense rate for methamphetamine				
Intercept (β_0_)	39.21	3.69	10.62	< .0001
Baseline slope (β_1_)	0.66	0.36	1.83	0.09
First-time offense rate for ecstasy				
Intercept (β_0_)	16.91	2.01	8.42	< .0001
Baseline slope (β_1_)	− 0.77	0.20	− 3.92	0.001

**Table 3:** Time series modeling of age-standardized first-time offense rates for heroin from 2001 to 2017 in Taiwan, separately for five age groups

Parameters in the parsimonious model	Coefficient estimate	Standard error	t‐statistic	*P* value
Age group 18–24 years old				
Intercept (β_0_)	14.31	0.76	18.85	< .0001
Baseline slope (β_1_)	− 0.51	0.12	− 4.24	0.001
Level change after intervention (β_2_)	− 6.17	1.30	− 4.76	0.00
β_2_/β_1_	12.10			
Age group 25–29 years old				
Intercept (β_0_)	19.54	1.12	17.51	< .0001
Baseline slope (β_1_)	− 0.75	0.18	− 4.24	0.001
Level change after intervention (β_2_)	− 7.97	1.91	− 4.18	0.00
β_2_/β_1_	10.63			
Age group 30–39 years old				
Intercept (β_0_)	27.76	1.82	15.24	< .0001
Baseline slope (β_1_)	− 1.26	0.29	− 4.35	0.001
Level change after intervention (β_2_)	− 7.06	3.11	− 2.27	0.04
β_2_/β_1_	5.60			
Age group 40–49 years old				
Intercept (β_0_)	11.64	0.95	12.22	< .0001
Baseline slope (β_1_)	− 0.59	0.15	− 3.88	0.002
Level change after intervention (β_2_)	− 1.27	1.63	− 0.78	0.45
Age group 50–59 years old				
Intercept (β_0_)	2.60	0.26	10.15	< .0001
Baseline slope (β_1_)	− 0.14	0.04	− 3.41	0.004
Level change after intervention (β_2_)	0.09	0.44	0.22	0.83

### Segmented regression analysis of the first-time offense rates

**Fig. 2:**
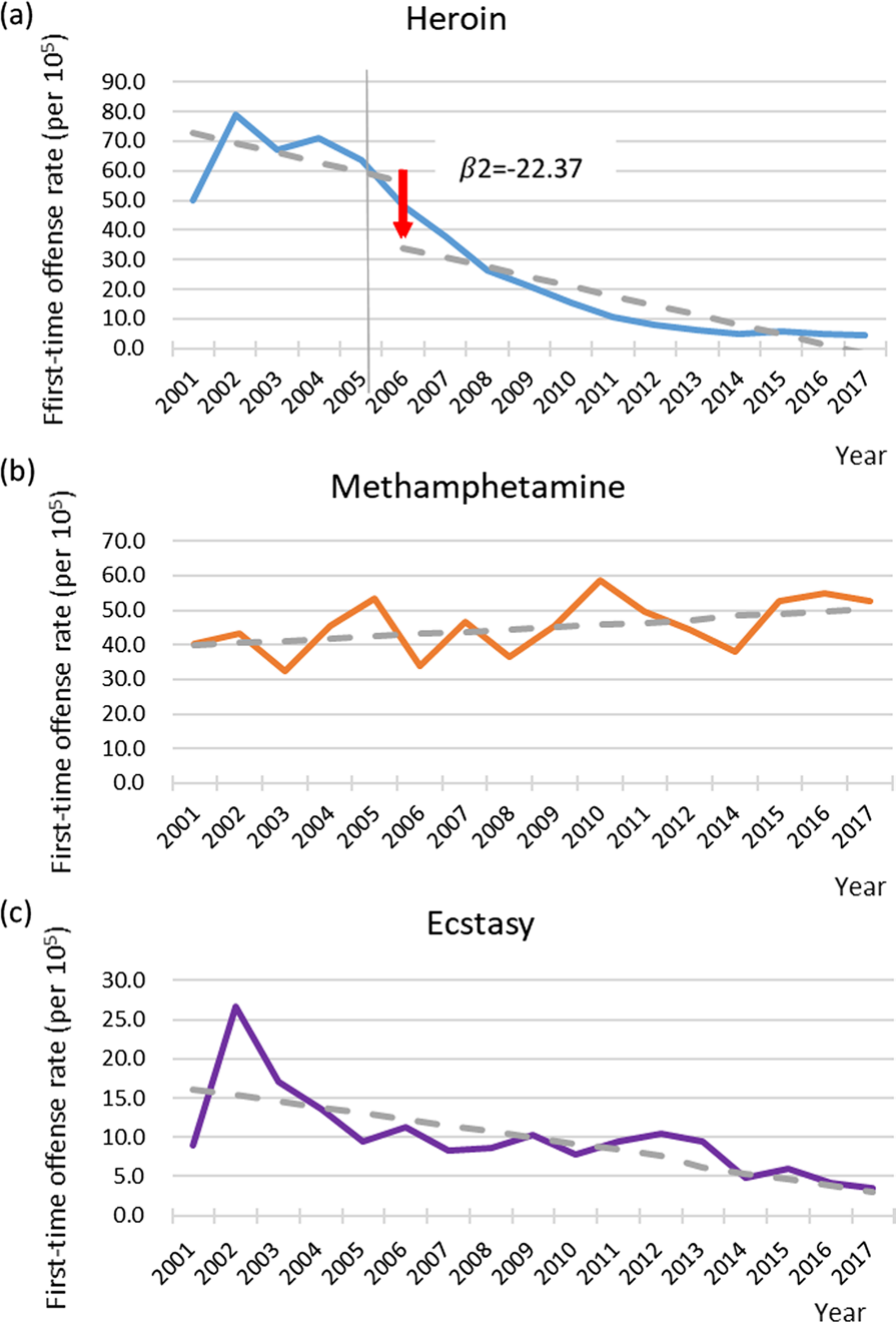
Yearly age-standardized first-time offense rate from 2001 to 2017 in Taiwan with the most parsimonious model in segmented regression analysis that had an intervention in 2006 for **a** heroin, with a three-parameter model; **b** methamphetamine, with a two-parameter model; and **c** ecstasy, with a two-parameter model

For the three most common categories of illicit drugs that tested positive prior to 2006, i.e., heroin, methamphetamine, and ecstasy, their age-standardized first-time offense rates were then subjected to segmented regression analysis to test for intervention effects. Initially, a four-parameter model consisting of β_0_, β_1_, β_2_, and β_3_ was fitted for each category of illicit drugs (detailed results are provided in Additional file 1: Table S3). After stepwise elimination of nonsignificant terms, the most parsimonious model was derived for each category of illicit drugs (Table 2). Only the parsimonious model of heroin had a significant level change (β_2_), whereas the parsimonious model of neither methamphetamine nor ecstasy had any significant estimate for β_2_ and β_3_. As shown in Fig. 2, after the intervention, there was a decrease (22.37 per 100,000; *P* = 0.01) in the first-time offense rate for heroin, which would take 6.88 years to accumulate by the baseline slope (− 3.25 per 100,000 per year; *P* = 0.001), though the slope remained the same after the intervention. In contrast, the first-time offense rate of methamphetamine had an increasing trend (0.66 per 100,000 per year; *P* = 0.09, borderline significance) and that of ecstasy had a decreasing trend (− 0.77 per 100,000 per year; *P* = 0.001) since 2001; neither rate was influenced by the intervention.

### Trend in the newly regulated recreational use of ketamine

In addition to the trends revealed in the segmented regression analysis for heroin, methamphetamine, and ecstasy, Fig. 1 further reveals a rapid rise in noncriminal use of ketamine after it was enrolled in the administrative penalty system in 2009; before that, only people who sold or manufactured ketamine (i.e., a criminal offense) would be arrested. The rate of noncriminal ketamine use first surpassed that of heroin in 2010, then surpassed that of methamphetamine in 2012, and peaked in 2013. After that, the rate of noncriminal use of ketamine started to decline steadily and substantially over the year, whereas that of methamphetamine use increased to a plateau.

**Fig. 3:**
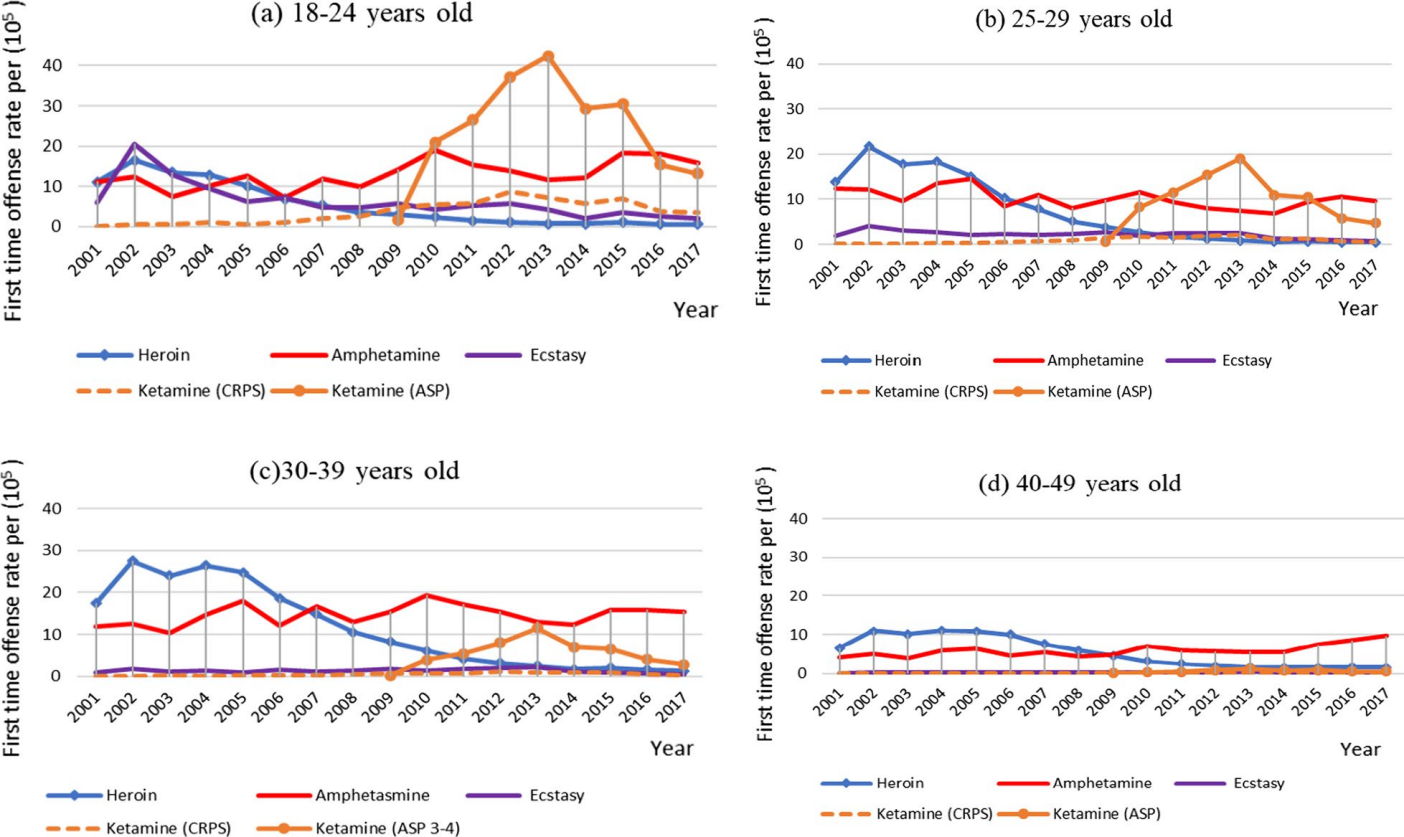
Yearly first-time offense rate from 2001 to 2017 for five hierarchically classified kinds of illicit drugs, including heroin, methamphetamine, ecstasy, ketamine-criminal prosecution, and ketamine-administrative penalty, in Taiwan from 2001 to 2017 separately for the age groups of **a** 18–24 years old, **b** 25–29 years old, **c** 30–39 years old, and **d** 40–49 years old

Next, we examined the yearly first-time offense rates of hierarchically classified drug categories separately for four age groups (Fig. 3). Of note, the corresponding figures for the group of 50–59 years old had little change and remained < 3% throughout the years and hence are not displayed in Fig. 3. Before 2006, heroin was the most commonly tested positive illicit drug for all age groups except the youngest one of 18–24 years old, in which methamphetamines and ecstasy were intertwined with heroin. Meanwhile, with the adoption of the administrative penalty against recreational use of ketamine in 2009, its first-time offense rates increased rapidly among the two youngest groups (18–24 and 25–29 years old), even surpassing that of methamphetamines. In contrast, for the oldest two groups (30–39, and 40–49 years old), the first-time offense rates for ketamine increased moderately but did not exceed that of methamphetamine for the group of 30–39 years old and remained extremely low for the oldest groups (40–49 years old). The relevant data for Fig. 3 are provided in Additional file 1: Table S4.

When the segmented regression model containing β_0_, β_1_, and β_2_ for heroin was applied to each of five age groups (Table 3), only the three youngest age groups (18–24, 25–29, and 30–39 years old) had a detectable level change (β_2_). Of note, the ratio of β_2_ to β_1_ was 12.10 for the 18–24-year-old group, decreased to 10.63 for the 25–29-year-old group and then to 5.6 for the 30–39-year-old group.

## Discussion

Using segmented regression analysis of the yearly first-time offense rates from 2001 to 2017, this study found that there was a decrease of 22.37 per 100,000 in the rate for heroin but no detectable level changes in that for methamphetamine or ecstasy after the 2006 nationwide implementation of the harm reduction program in Taiwan. Additionally, there were decreasing trends in the first-time offense rate from 2001 to 2017 for heroin and ecstasy and an increasing trend for methamphetamine, with the slopes not altered by the 2006 intervention. When examined in five different age groups, the postintervention decrease in the first-time offense rate for heroin was detectable among the youngest three groups (18–24, 25–29, and 30–39 years old) but not seen for the groups of 40–49 and 50–59 years old. Additionally, the newly regulated recreational use of ketamine since the end of 2009 quickly gained popularity and became the most common illicit drug that tested positive among first-time offenders younger than 30 years old. Our results provide empirical support for a diffusion effect of the 2006 intervention on decreasing heroin use among young offenders and have policy implications for better prevention and treatment for different age groups.

The evaluation whether drug users' choice of illicit drugs was influenced by an intervention, such as the implementation of a nationwide harm reduction program, presents several methodological challenges. The first challenge is the lack of reliable epidemiological estimates of unsanctioned drug use [28]. In this aspect, the drug arrest data that have been verified using urine tests can be helpful [29]. The second challenge is the distinction of first-time drug offenders from repeat drug offenders, since repeat offenders’ choice of drugs might be driven by their craving for the drug of their current use. In a previous study relying on the results of the indicated urine tests, which did not separate repeated offenses from first-time offenses, heroin use did not decrease in the first three years after the implementation of a harm reduction program [13]. It remains unknown whether the choice of illicit drugs among first-time offenders was different from that among repeat offenders. The third challenge is that alterations in the preferred categories of illicit drugs might be influenced by other concurrent changes. For example, the emergence of so-called club drugs [30] or party drugs [31] in the early 2000s led to substantial changes in the landscape of illicit drug use, especially among young people, in many countries, including Taiwan [32, 33]. To meet these challenges, time series data that can be subjected to segmented regression analysis are needed for the evaluation of the impact of an intervention [21, 22, 27].

The feasibility of our analyses of the first-time offenses for illicit drugs was mainly based on a combination of the availability of various information, including the verification of drug use by urine tests, national drug enforcement databases that included all illicit drug offenses, and a comprehensive listing of illicit drugs tested positive in each arrest. By means of record linkage without revealing the identity of people arrested for illicit drug offense, we were able to trace an individual’s illicit drug offense to its first appearance and assign all the offenses in other years as repeat offenses. Owing to the long period (2001–2017) covered by these databases, we could apply segmented regression to quantitatively evaluate the impact of the 2006 intervention on heroin use among first-time offenders.

Under the traditional harsh punishment stipulated in antinarcotic laws in Asia [8], many countries in this region have been reluctant to adopt harm reduction programs to tackle the increase in the incidence of HIV [4]. After the Taiwanese government responded to the spike of the HIV epidemic by adopting the three-pronged harm reduction program in 2006, outcome evaluations were focused on the remarkable decline in HIV incidence among people who injected drugs [13, 15, 34]. For the first time, this study provides new insight into the discouraging or diffusion effect (Clarke and Weisburd, 1994; Guerette, 2009) of the harm reduction program on heroin use among first-time illicit drug offenders.

Based on the yearly first-time offense rates for individual categories of hierarchically classified illicit drugs derived in this study, heroin was the most common drug that tested positive among the first-time illicit drug offenders in the early 2000s. One explanation is that individuals with a first arrest for an illicit drug offense (heroin) might have used other illicit drugs previously. On the other hand, this pattern is compatible with the finding of a study of youths aged 15 to 22 incarcerated in 2003 for illicit drug use in Northern Taiwan in which 54% of the offenders were heroin users, of whom 83% had IDU [35].

The results of segmented regression analysis revealed that among the three most commonly used illicit drugs prior to the 2006 intervention, only heroin exhibited a postintervention decrease in the first-time offense rate. It appears that the diffusion effect of the nationwide harm reduction program was specific to drugs administered mainly via injection. Additionally, all three drugs had a baseline trend, i.e., a decreasing trend for heroin and ecstasy and an increasing trend for methamphetamine. Under this circumstance, the intervention-induced decrease of 22.37 per 100,000 in the first-time offense rate for heroin was equivalent to a drop accumulated over 6.88 years. In other words, despite a decreasing trend in the first-time offense rate for heroin that was already in place in the early 2000s, the 2006 intervention did help to accelerate the decline in heroin use within a year.

Furthermore, the intervention-induced decrease in heroin use was found to be greater for younger age groups. Hence, this diffusion effect on heroin use is likely to be associated with changes in drug use culture among young people. It is plausible that the hazard of contracting HIV by means of IDU might have spread more easily to the community of younger drug users and prevented them from using heroin. Some anecdotal reports indicate that young drug offenders tended to think of heroin injection as not only hazardous for contracting HIV but also as being “old-fashioned.”

Nevertheless, an intervention for IDU might also lead to crime displacement [36]. There was a growing popularity of party or club drugs among young people [32, 33]. Examining the first-time offense rates for individual categories of illicit drugs during the study period, it was found that ketamine increased steadily from its enrollment in late 2009 and became the most common drug that tested positive in 2013, especially among offenders younger than 30 years old.

The popularity of ketamine use was initially noted after another rave-associated drug, ecstasy, was elevated to Schedule II in 1999 [11], which accounted for the decreasing trend of ecstasy use since the beginning of the period covered by this study. Drug dealers began to promote ketamine over ecstasy because ketamine consumption would not lead to incarceration [32]. Following a legal amendment in 2009, even noncriminal possession of ketamine (i.e., < 20 g) was officially outlawed, and its proportion among young adult first-time offenders surged quickly and overtook the leading role of methamphetamine among young adults (18–24 and 25–29 age groups). Intriguingly, the popularity of ketamine began to drop after reaching its peak in 2013. A likely explanation is that the price of ketamine became higher after a large number of clandestine ketamine laboratories (118 in 2013 and 89 in 2014) were dismantled in China, a main source of Taiwan’s ketamine, and indeed, the quantities of ketamine seized also steadily increased globally from 2013 to 2015 [37] and locally from 2013 to 2014 [38]. This phenomenon highlights the potential influence of market availability on illicit drug use, similar to the attribution of the recent re-emergence of HIV among people with IDU in many European countries to cocaine injection [39, 40] as indicated by changes in Europe’s cocaine market [41].

To a lesser extent, methamphetamine might also contribute to a small proportion of the decline in heroin use among first-time illicit drug offenders, since it had a borderline increasing trend at baseline. Notably, methamphetamine, the predominant illicit drug in the 1990s [11], has re-emerged as the top drug of choice among first-time offenders since 2015, following the decline of recreational use of ketamine in 2014. This is compatible with a recent urinalysis study among patients in Taiwan’s emergency departments in 2017 and 2018, in which patients suspected of drug intoxication were ordered to undergo toxicological screening [42]. Among the illicit substances identified by liquid chromatography/tandem mass spectrometry, methamphetamine (67%) was the most frequently identified, followed by ketamine (21.7%) and opioids (15.8%). Furthermore, in the three waves of Taiwan’s national survey of substance use in 2005, 2009, and 2014, methamphetamine persisted as the most frequently used illicit drug [43]. The long-standing popularity of methamphetamine is probably attributed partly to the fact that the drug can be easily made by transnational criminal organizations in small clandestine laboratories, with relatively inexpensive over-the-counter ingredients. This is supported by the observation that since 1998, the largest quantity of amphetamine-type stimulants seized was methamphetamine, accounting for 66% of global seizures over the period 2013–2017 [44].

Taken together, the nationwide implementation of harm reduction program in 2006 did succeed in reducing the incidence of HIV infection among people with IDU and decreasing heroin use among first-time offenders as well. Nevertheless, this does not necessarily imply it would be equally effective in the control of HIV transmission due to other risk factors [39]. In fact, a separate sexually transmitted HIV outbreak involving mainly men who have sex with men (MSM) continued to rise exponentially since 2006 in Taiwan [45–47]. Accompanying this, there appeared to be an increasing trend of recreational drug use among MSM [48]. An online anonymous survey from December 2018 to January 2019 among participants of a MSM social network found that 24.7% of respondents reported injection of methamphetamine within the past 6 months [49]. Furthermore, MSM were found to have increased risk of non-opioid recreational drug use [50]. This indicates that a new combination of IDU with methamphetamine in MSM may constitute a future challenge in the control of both HIV and illicit drug use.

### Implications

Our findings have implications for the control policy of illicit drug use. Many countries in Asia have been hesitant to implement harm reduction responses when facing HIV epidemics among people with IDU [8–10]. One concern was that such an approach might have implicit consent for IDU and could lead to an increase in heroin use. Our results indicate that the implementation of a nationwide harm reduction program not only led to a remarkable decline in HIV incidence among people with IDU but also had a diffusion effect on decreasing heroin use among first-time illicit drug offenders.

Nevertheless, our results also revealed that the decline in the first-time offense rate for heroin was partly offset by the increase in the first-time offense rate for methamphetamine and ketamine. Given that the recidivism and mortality rate of both methamphetamine [51] and ketamine [23] have been found to be high, this poses new challenges for their prevention and treatment. A further warning about the re-emergence of methamphetamine is that it has become a new drug of choice for injection use among MSM [49]. New approaches, such as a combination of both medical and psychosocial interventions of sufficient intensity [13], warrant further development to alleviate the surge of recreational use of party drugs.

### Limitations

This study has several limitations. First, the enforcement of laws related to illicit drug use might be influenced by factors unrelated to drug regulation. Second, the drug arrest data did not have information on one’s pattern of use, motivation for use, and drug sources. Hence, we do not know about injection patterns or motivation for such use. Third, we do not have symptom or comorbidity information. Fourth, the current databases do not contain other criminal record data. Finally, this study did not have information about drug market changes over time. To what extent the change in certain first-time illicit drug use could be accounted for by market availability remains unknown.

## Conclusion

In summary, the 2006 intervention via nationwide implementation of a harm reduction program helped to accelerate the drop in heroin use among young first-time illicit drug offenders. Before 2013, ketamine seemed to be a substitute for heroin in this age group. However, after the surge in ketamine’s price, methamphetamine re-emerged as the most common illicit drug used by first-time offenders. Further investigation into evidence-based alternative ways to prevent and treat the use of specific categories of illicit drugs is urgently needed.

## Supplementary Information

**Additional file 1**. **Table S1.** Number of illicit drug offences (in events), from schedule I to IV, and offenders (in persons), aged 18–69, 2001–2017, Taiwan. **Table S2.** Number of first-time illicit drug offenders in Taiwan, 2001–2017, stratified by sex and age groups. **Table S3.** Time series modeling with segmented regression that contains four parameters of age-standardized first-time offence rates (per 100,000) for heroin, methamphetamine, and ecstasy, respectively, from 2001 to 2017 in Taiwan. **Table S4.** Age-standardized first-time offence rate for hierarchically classified illicit drugs in Taiwan, 2001–2017, stratified by age groups: (a) 18–24 years old; (b) 25–29 years old; (c) 30–39 years old; (d) 40–49 years old; and (e) 50–59 years old.

## Data Availability

The datasets analyzed during the current study are not publicly available due to the requirement of obtaining official permission to access the data, but are available from the corresponding author on reasonable request.

## Abbreviations

APS: Administrative Penalty System for Schedule III/IV Substances
CRPS: Criminal Record Processing System
HIV: Human immunodeficiency virus
IDU: Injection drug use
