# Supplementary material for: The impact of Taiwan’s implementation of a nationwide harm reduction program in 2006 on the use of various illicit drugs: trend analysis of first-time offenders from 2001 to 2017
Source: Harm Reduct J. 2021 Nov 19;18:117. doi: 10.1186/s12954-021-00566-5 (PMC8603590; doi:10.1186/s12954-021-00566-5)
Supplement: Supplementary file 1 — Additional file 1. Table S1. Number of illicit drug offences (in events), from schedule I to IV, and offenders (in persons), aged 18–69, 2001–2017, Taiwan. Table S2. Number of first-time illicit drug offenders in Taiwan, 2001–2017, stratified by sex and age groups. Table S3. Time series modeling with segmented regression that contains four parameters of age-standardized first-time offence rates (per 100,000) for heroin, methamphetamine, and ecstasy, respectively, from 2001 to 2017 in Taiwan. Table S4. Age-standardized first-time offence rate for hierarchically classified illicit drugs in Taiwan, 2001–2017, stratified by age groups: (a) 18–24 years old; (b) 25–29 years old; (c) 30–39 years old; (d) 40–49 years old; and (e) 50–59 years old. [file 12954_2021_566_MOESM1_ESM.doc]

**Supplementary material (Online-only)**

**Table S1**. Number of illicit drug offences (in events), from schedule I to IV, and offenders (in persons), aged 18 - 69, 2001-2017, Taiwan.

**Table S2**. Number of first-time illicit drug offenders in Taiwan, 2001-2017, stratified by sex and age groups.

**Table S3**. Time series modeling with segmented regression that contains four parameters of age-standardized first-time offence rates (per 100,000) for heroin, methamphetamine, and ecstasy, respectively, from 2001 to 2017 in Taiwan.

**Table S4.1** Age-standardized first-time offence rate for hierarchically classified illicit drugs in Taiwan, 2001-2017, stratified by age groups: (a) 18-24 years old.

**Table S4.2** Age-standardized first-time offence rate for hierarchically classified illicit drugs in Taiwan, 2001-2017, stratified by age groups: (b) 25-29 years old.

**Table S4.3** Age-standardized first-time offence rate for hierarchically classified illicit drugs in Taiwan, 2001-2017, stratified by age groups: (c) 30-39 years old.

**Table S4.4** Age-standardized first-time offence rate for hierarchically classified illicit drugs in Taiwan, 2001-2017, stratified by age groups: (d) 40-49 years old.

**Table S4.5** Age-standardized first-time offence rate for hierarchically classified illicit drugs in Taiwan, 2001-2017, stratified by age groups: (e) 50-59 years old.

Table S1. Number of illicit drug offences (in events), from schedule I to IV, and offenders (in persons), aged 18 - 69, 2001-2017, Taiwan.

|  | Population  (age 18 to 69) | | Drug offence  (event) | |  | Drug offender  (person) | |  | First-time  drug offender | |  | Event per  offender |  | % of first-time offenders | Age-standardized rate^a^  (per 1000) | | |
| --- | --- | --- | --- | --- | --- | --- | --- | --- | --- | --- | --- | --- | --- | --- | --- | --- | --- |
| Year | N |  | N_1_ | Crude (‰) |  | N_2_ | Crude (‰) |  | N_3_ | Crude (‰) |  | N_1/_N_2_ |  | N_3_/N_2_ (%) |  | Offenders | First-time offenders |
| 2001 | 15425717 | | 16723 | 1.1 |  | 14255 | 0.9 |  | 14255 | 0.9 |  | 1.17 |  | 100.0 | 0.91 | | 0.91 |
| 2002 | 15610445 | | 30527 | 2.0 |  | 24628 | 1.6 |  | 21423 | 1.4 |  | 1.24 |  | 87.0 | 2.01 | | 1.73 |
| 2003 | 15764055 | | 28961 | 1.8 |  | 22890 | 1.5 |  | 16963 | 1.1 |  | 1.27 |  | 74.1 | 1.87 | | 1.37 |
| 2004 | 15887992 | | 41734 | 2.6 |  | 28572 | 1.8 |  | 18974 | 1.2 |  | 1.46 |  | 66.4 | 2.34 | | 1.54 |
| 2005 | 16025913 | | 53115 | 3.3 |  | 34159 | 2.1 |  | 18347 | 1.1 |  | 1.55 |  | 53.7 | 2.74 | | 1.47 |
| 2006 | 16219510 | | 48740 | 3.0 |  | 30879 | 1.9 |  | 13885 | 0.9 |  | 1.58 |  | 45.0 | 2.39 | | 1.08 |
| 2007 | 16362008 | | 56491 | 3.5 |  | 34653 | 2.1 |  | 13974 | 0.9 |  | 1.63 |  | 40.3 | 2.65 | | 1.09 |
| 2008 | 16529026 | | 53817 | 3.3 |  | 33238 | 2.0 |  | 11033 | 0.7 |  | 1.62 |  | 33.2 | 2.46 | | 0.86 |
| 2009 | 16681040 | | 48646 | 2.9 |  | 31759 | 1.9 |  | 12176 | 0.7 |  | 1.53 |  | 38.3 | 2.36 | | 0.97 |
| 2010 | 16815313 | | 60018 | 3.6 |  | 38653 | 2.3 |  | 16538 | 1.0 |  | 1.55 |  | 42.8 | 2.96 | | 1.37 |
| 2011 | 16951851 | | 58931 | 3.5 |  | 38861 | 2.3 |  | 15976 | 0.9 |  | 1.52 |  | 41.1 | 3.02 | | 1.35 |
| 2012 | 17082947 | | 62230 | 3.6 |  | 41556 | 2.4 |  | 17201 | 1.0 |  | 1.50 |  | 41.4 | 3.29 | | 1.49 |
| 2013 | 17212889 | | 65290 | 3.8 |  | 43714 | 2.5 |  | 17656 | 1.0 |  | 1.49 |  | 40.4 | 3.55 | | 1.55 |
| 2014 | 17341152 | | 57288 | 3.3 |  | 38747 | 2.2 |  | 13186 | 0.8 |  | 1.48 |  | 34.0 | 3.08 | | 1.12 |
| 2015 | 17487152 | | 70684 | 4.0 |  | 45284 | 2.6 |  | 15493 | 0.9 |  | 1.56 |  | 34.2 | 3.57 | | 1.31 |
| 2016 | 17564730 | | 69065 | 3.9 |  | 43164 | 2.5 |  | 12382 | 0.7 |  | 1.60 |  | 28.7 | 3.33 | | 1.04 |
| 2017 | 17624708 | | 67461 | 3.8 |  | 41638 | 2.4 |  | 11623 | 0.7 |  | 1.62 |  | 27.9 | 3.21 | | 0.97 |

^a^ using the World Standard population (WHO 2000-2025) (Ahmad et al., 2001), truncated to the age range between 18 to 69 years, as the weighting for the population (18-24 years: 18.24%; 25-29 years: 12.49%; 30-39 years: 23.24%; 40-49 years: 19.89%; 50-59 years: 15.62%; and 60-69 years: 10.52%).

Table S2. Number of first-time illicit drug offenders in Taiwan, 2001-2017, stratified by sex and age groups.

|  |  | Sex | | |  | Age groups (years) | | | | | |
| --- | --- | --- | --- | --- | --- | --- | --- | --- | --- | --- | --- |
| Year | Total | Male | (%) | Female |  | 18-24 | 25-29 | 30-39 | 40-49 | 50-59 | 60-69 |
| 2001 | 14255 | 12208 | (85.6) | 2047 |  | 3912 | 3742 | 4591 | 1685 | 270 | 55 |
| 2002 | 21423 | 18078 | (84.4) | 3345 |  | 6772 | 5206 | 6326 | 2599 | 458 | 62 |
| 2003 | 16963 | 14226 | (83.9) | 2737 |  | 4575 | 4253 | 5295 | 2339 | 446 | 55 |
| 2004 | 18974 | 16001 | (84.3) | 2973 |  | 4360 | 4929 | 6249 | 2807 | 555 | 74 |
| 2005 | 18347 | 15292 | (83.3) | 3055 |  | 3647 | 4666 | 6336 | 2939 | 679 | 80 |
| 2006 | 13884 | 11376 | (81.9) | 2508 |  | 2691 | 3223 | 4749 | 2520 | 649 | 52 |
| 2007 | 13974 | 11632 | (83.2) | 2342 |  | 2806 | 3239 | 4878 | 2282 | 693 | 76 |
| 2008 | 11034 | 9147 | (82.9) | 1887 |  | 2375 | 2465 | 3789 | 1820 | 520 | 65 |
| 2009 | 12176 | 10170 | (83.5) | 2006 |  | 3222 | 2689 | 3959 | 1657 | 572 | 77 |
| 2010 | 16538 | 13631 | (82.4) | 2907 |  | 5650 | 3680 | 4763 | 1811 | 571 | 63 |
| 2011 | 15976 | 13062 | (81.8) | 2914 |  | 5826 | 3539 | 4460 | 1564 | 520 | 67 |
| 2012 | 17201 | 14149 | (82.3) | 3052 |  | 7132 | 3604 | 4498 | 1468 | 434 | 65 |
| 2013 | 17656 | 14524 | (82.3) | 3139 |  | 7195 | 3834 | 4638 | 1483 | 424 | 82 |
| 2014 | 13186 | 10917 | (82.8) | 2268 |  | 5392 | 2378 | 3584 | 1348 | 416 | 68 |
| 2015 | 15493 | 12982 | (83.8) | 2504 |  | 6503 | 2598 | 4094 | 1637 | 570 | 91 |
| 2016 | 12382 | 10337 | (83.5) | 2045 |  | 4315 | 2142 | 3435 | 1820 | 559 | 111 |
| 2017 | 11623 | 9673 | (83.2) | 1950 |  | 3789 | 1938 | 3155 | 1946 | 656 | 139 |

| Table S3. Time series modeling with segmented regression that contains four parameters of age-standardized first-time offence rates (per 100,000) for heroin, methamphetamine, and ecstasy, respectively, from 2001 to 2017 in Taiwan. | | | | |
| --- | --- | --- | --- | --- |
| Parameters | Coefficient estimate | Standard error | t‐statistic | P‐value |
| First-time offence rate for heroin |  |  |  |  |
| Intercept (β_0_) | 48.09 | 16.15 | 2.98 | 0.01 |
| Baseline slope (β_1_) | 8.66 | 7.47 | 1.16 | 0.27 |
| Level change after intervention (β_2_) | -13.32 | 11.35 | -1.17 | 0.26 |
| Slope change after intervention (β_3_) | -13.64 | 7.51 | -1.82 | 0.09 |
| First-time offence rate for methamphetamine |  |  |  |  |
| Intercept (β_0_) | 46.47 | 11.46 | 4.06 | 0.00 |
| Baseline slope (β_1_) | -3.94 | 5.30 | -0.74 | 0.47 |
| Level change after intervention (β_2_) | 8.26 | 8.05 | 1.03 | 0.32 |
| Slope change after intervention (β_3_) | 4.42 | 5.33 | 0.83 | 0.42 |
| First-time offence rate for ecstasy |  |  |  |  |
| Intercept (β_0_) | 9.43 | 5.38 | 1.75 | 0.10 |
| Baseline slope (β_1_) | 4.07 | 2.49 | 1.64 | 0.13 |
| Level change after intervention (β_2_) | -9.05 | 3.78 | -2.40 | 0.03 |
| Slope change after intervention (β_3_) | -4.63 | 2.50 | -1.85 | 0.09 |

Table S4.1 Age-standardized first-time offence rate for hierarchically classified illicit drugs in Taiwan, 2001-2017, stratified by age groups

(a) 18-24 years old.

| Year | Total | Heroin | |  | Methamphetamine | |  | Ecstasy | |  | Ketamine (CRPS) | |  | Ketamine (APS) | |  | Others | |
| --- | --- | --- | --- | --- | --- | --- | --- | --- | --- | --- | --- | --- | --- | --- | --- | --- | --- | --- |
|  |  | N | (%) |  | N | % |  | N | % |  | N | % |  | N | % |  | N | % |
| 2001 | 3912 | 1527 | (39.03) |  | 1487 | (38.01) |  | 792 | (20.25) |  | 0 | (0.00) |  | - | - |  | 106 | (2.71) |
| 2002 | 6772 | 2210 | (32.63) |  | 1636 | (24.16) |  | 2678 | (39.55) |  | 74 | (1.09) |  | - | - |  | 174 | (2.57) |
| 2003 | 4575 | 1775 | (38.80) |  | 951 | (20.79) |  | 1638 | (35.80) |  | 68 | (1.49) |  | - | - |  | 143 | (3.13) |
| 2004 | 4360 | 1645 | (37.73) |  | 1264 | (28.99) |  | 1163 | (26.67) |  | 142 | (3.26) |  | - | - |  | 146 | (3.35) |
| 2005 | 3647 | 1257 | (34.47) |  | 1502 | (41.18) |  | 738 | (20.24) |  | 76 | (2.08) |  | - | - |  | 74 | (2.03) |
| 2006 | 2691 | 821 | (30.51) |  | 844 | (31.36) |  | 831 | (30.88) |  | 110 | (4.09) |  | - | - |  | 85 | (3.16) |
| 2007 | 2806 | 626 | (22.31) |  | 1336 | (47.61) |  | 539 | (19.21) |  | 214 | (7.63) |  | - | - |  | 91 | (3.24) |
| 2008 | 2375 | 391 | (16.46) |  | 1093 | (46.02) |  | 515 | (21.68) |  | 281 | (11.83) |  | - | - |  | 95 | (4.00) |
| 2009 | 3222 | 321 | (9.96) |  | 1532 | (47.55) |  | 608 | (18.87) |  | 511 | (15.86) |  | 174 | (5.40) |  | 76 | (2.36) |
| 2010 | 5650 | 248 | (4.39) |  | 2053 | (36.34) |  | 422 | (7.47) |  | 585 | (10.35) |  | 2261 | (40.02) |  | 81 | (1.43) |
| 2011 | 5826 | 168 | (2.88) |  | 1659 | (28.48) |  | 514 | (8.82) |  | 605 | (10.38) |  | 2843 | (48.80) |  | 37 | (0.64) |
| 2012 | 7132 | 128 | (1.79) |  | 1539 | (21.58) |  | 545 | (7.64) |  | 929 | (13.03) |  | 3916 | (54.91) |  | 75 | (1.05) |
| 2013 | 7195 | 82 | (1.14) |  | 1295 | (18.00) |  | 425 | (5.91) |  | 788 | (10.95) |  | 4513 | (62.72) |  | 92 | (1.28) |
| 2014 | 5392 | 105 | (1.95) |  | 1299 | (24.09) |  | 217 | (4.02) |  | 638 | (11.83) |  | 3094 | (57.38) |  | 39 | (0.72) |
| 2015 | 6503 | 138 | (2.12) |  | 1965 | (30.22) |  | 367 | (5.64) |  | 749 | (11.52) |  | 3205 | (49.28) |  | 79 | (1.21) |
| 2016 | 4315 | 66 | (1.53) |  | 1914 | (44.36) |  | 252 | (5.84) |  | 398 | (9.22) |  | 1556 | (36.06) |  | 129 | (2.99) |
| 2017 | 3789 | 69 | (1.82) |  | 1654 | (43.65) |  | 214 | (5.65) |  | 378 | (9.98) |  | 1241 | (32.75) |  | 233 | (6.15) |

Table S4.2 Age-standardized first-time offence rate for hierarchically classified illicit drugs in Taiwan, 2001-2017, stratified by age groups

(b) 25-29 years old.

| Year | Total | Heroin | |  | Methamphetamine | |  | Ecstasy | |  | Ketamine (CRPS) | |  | Ketamine (APS) | |  | Others | |
| --- | --- | --- | --- | --- | --- | --- | --- | --- | --- | --- | --- | --- | --- | --- | --- | --- | --- | --- |
|  |  | N | % |  | N | % |  | N | % |  | N | % |  | N | % |  | N | % |
| 2001 | 3742 | 1875 | (50.11) |  | 1585 | (42.36) |  | 237 | (6.33) |  | 0 | (0.00) |  | - | - |  | 45 | (1.20) |
| 2002 | 5206 | 2962 | (56.90) |  | 1589 | (30.52) |  | 542 | (10.41) |  | 24 | (0.46) |  | - | - |  | 89 | (1.71) |
| 2003 | 4253 | 2441 | (57.39) |  | 1279 | (30.07) |  | 418 | (9.83) |  | 24 | (0.56) |  | - | - |  | 91 | (2.14) |
| 2004 | 4929 | 2577 | (52.28) |  | 1858 | (37.70) |  | 371 | (7.53) |  | 32 | (0.65) |  | - | - |  | 91 | (1.85) |
| 2005 | 4666 | 2217 | (47.51) |  | 2066 | (44.28) |  | 278 | (5.96) |  | 37 | (0.79) |  | - | - |  | 68 | (1.46) |
| 2006 | 3223 | 1519 | (47.13) |  | 1209 | (37.51) |  | 338 | (10.49) |  | 78 | (2.42) |  | - | - |  | 79 | (2.45) |
| 2007 | 3239 | 1188 | (36.68) |  | 1571 | (48.50) |  | 304 | (9.39) |  | 104 | (3.21) |  | - | - |  | 72 | (2.22) |
| 2008 | 2465 | 763 | (30.95) |  | 1165 | (47.26) |  | 320 | (12.98) |  | 127 | (5.15) |  | - | - |  | 90 | (3.65) |
| 2009 | 2689 | 558 | (20.75) |  | 1391 | (51.73) |  | 371 | (13.80) |  | 213 | (7.92) |  | 79 | (2.94) |  | 77 | (2.86) |
| 2010 | 3680 | 380 | (10.33) |  | 1597 | (43.40) |  | 253 | (6.88) |  | 228 | (6.20) |  | 1160 | (31.52) |  | 62 | (1.68) |
| 2011 | 3539 | 233 | (6.58) |  | 1252 | (35.38) |  | 310 | (8.76) |  | 205 | (5.79) |  | 1501 | (42.41) |  | 38 | (1.07) |
| 2012 | 3604 | 156 | (4.33) |  | 1007 | (27.94) |  | 284 | (7.88) |  | 247 | (6.85) |  | 1865 | (51.75) |  | 45 | (1.25) |
| 2013 | 3834 | 102 | (2.66) |  | 910 | (23.74) |  | 265 | (6.91) |  | 250 | (6.52) |  | 2251 | (58.71) |  | 56 | (1.46) |
| 2014 | 2378 | 60 | (2.52) |  | 784 | (32.97) |  | 136 | (5.72) |  | 131 | (5.51) |  | 1235 | (51.93) |  | 32 | (1.35) |
| 2015 | 2598 | 70 | (2.69) |  | 1060 | (40.80) |  | 122 | (4.70) |  | 145 | (5.58) |  | 1153 | (44.38) |  | 48 | (1.85) |
| 2016 | 2142 | 59 | (2.75) |  | 1199 | (55.98) |  | 97 | (4.53) |  | 76 | (3.55) |  | 621 | (28.99) |  | 90 | (4.20) |
| 2017 | 1938 | 52 | (2.68) |  | 1103 | (56.91) |  | 84 | (4.33) |  | 67 | (3.46) |  | 503 | (25.95) |  | 129 | (6.66) |

Table S4.3 Age-standardized first-time offence rate for hierarchically classified illicit drugs in Taiwan, 2001-2017, stratified by age groups

(c) 30-39 years old.

| Year | Total | Heroin | |  | Methamphetamine | |  | Ecstasy | |  | Ketamine (CRPS) | |  | Ketamine (APS) | |  | Others | |
| --- | --- | --- | --- | --- | --- | --- | --- | --- | --- | --- | --- | --- | --- | --- | --- | --- | --- | --- |
|  |  | N | % |  | N | % |  | N | % |  | N | % |  | N | % |  | N | % |
| 2001 | 4591 | 2691 | (58.61) |  | 1721 | (37.49) |  | 143 | (3.11) |  | 0 | (0.00) |  | - | - |  | 36 | (0.78) |
| 2002 | 6326 | 4170 | (65.92) |  | 1818 | (28.74) |  | 265 | (4.19) |  | 8 | (0.13) |  | - | - |  | 65 | (1.03) |
| 2003 | 5295 | 3540 | (66.86) |  | 1502 | (28.37) |  | 148 | (2.80) |  | 34 | (0.64) |  | - | - |  | 71 | (1.34) |
| 2004 | 6249 | 3868 | (61.90) |  | 2117 | (33.88) |  | 191 | (3.06) |  | 18 | (0.29) |  | - | - |  | 55 | (0.88) |
| 2005 | 6336 | 3571 | (56.36) |  | 2554 | (40.31) |  | 141 | (2.23) |  | 26 | (0.41) |  | - | - |  | 44 | (0.69) |
| 2006 | 4749 | 2745 | (57.80) |  | 1719 | (36.20) |  | 207 | (4.36) |  | 21 | (0.44) |  | - | - |  | 57 | (1.20) |
| 2007 | 4878 | 2239 | (45.90) |  | 2364 | (48.46) |  | 161 | (3.30) |  | 48 | (0.98) |  | - | - |  | 66 | (1.35) |
| 2008 | 3789 | 1619 | (42.73) |  | 1849 | (48.80) |  | 193 | (5.09) |  | 55 | (1.45) |  | - | - |  | 73 | (1.93) |
| 2009 | 3959 | 1257 | (31.75) |  | 2246 | (56.73) |  | 256 | (6.47) |  | 98 | (2.48) |  | 33 | (0.83) |  | 69 | (1.74) |
| 2010 | 4763 | 953 | (20.01) |  | 2853 | (59.90) |  | 191 | (4.01) |  | 110 | (2.31) |  | 585 | (12.28) |  | 71 | (1.49) |
| 2011 | 4460 | 679 | (15.22) |  | 2543 | (57.02) |  | 244 | (5.47) |  | 116 | (2.60) |  | 810 | (18.16) |  | 68 | (1.52) |
| 2012 | 4498 | 508 | (11.29) |  | 2328 | (51.76) |  | 267 | (5.94) |  | 160 | (3.56) |  | 1166 | (25.92) |  | 69 | (1.53) |
| 2013 | 4638 | 411 | (8.86) |  | 1976 | (42.60) |  | 307 | (6.62) |  | 158 | (3.41) |  | 1710 | (36.87) |  | 76 | (1.64) |
| 2014 | 3584 | 286 | (7.98) |  | 1887 | (52.65) |  | 166 | (4.63) |  | 129 | (3.60) |  | 1048 | (29.24) |  | 68 | (1.90) |
| 2015 | 4094 | 339 | (8.28) |  | 2413 | (58.94) |  | 141 | (3.44) |  | 140 | (3.42) |  | 976 | (23.84) |  | 85 | (2.08) |
| 2016 | 3435 | 252 | (7.34) |  | 2354 | (68.53) |  | 108 | (3.14) |  | 61 | (1.78) |  | 560 | (16.30) |  | 100 | (2.91) |
| 2017 | 3155 | 218 | (6.91) |  | 2250 | (71.32) |  | 81 | (2.57) |  | 53 | (1.68) |  | 370 | (11.73) |  | 183 | (5.80) |

Table S4.4 Age-standardized first-time offence rate for hierarchically classified illicit drugs in Taiwan, 2001-2017, stratified by age groups

(d) 40-49 years old.

| Year | Total | Heroin | |  | Methamphetamine | |  | Ecstasy | |  | Ketamine (CRPS) | |  | Ketamine (APS) | |  | Others | |
| --- | --- | --- | --- | --- | --- | --- | --- | --- | --- | --- | --- | --- | --- | --- | --- | --- | --- | --- |
|  |  | N | % |  | N | % |  | N | % |  | N | % |  | N | % |  | N | % |
| 2001 | 1685 | 1038 | (61.60) |  | 625 | (37.09) |  | 13 | (0.77) |  | 0 | (0.00) |  | - | - |  | 9 | (0.53) |
| 2002 | 2599 | 1748 | (67.26) |  | 787 | (30.28) |  | 40 | (1.54) |  | 22 | (0.85) |  | - | - |  | 2 | (0.08) |
| 2003 | 2339 | 1641 | (70.16) |  | 631 | (26.98) |  | 36 | (1.54) |  | 7 | (0.30) |  | - | - |  | 24 | (1.03) |
| 2004 | 2807 | 1794 | (63.91) |  | 951 | (33.88) |  | 35 | (1.25) |  | 6 | (0.21) |  | - | - |  | 21 | (0.75) |
| 2005 | 2939 | 1810 | (61.59) |  | 1058 | (36.00) |  | 40 | (1.36) |  | 6 | (0.20) |  | - | - |  | 25 | (0.85) |
| 2006 | 2520 | 1692 | (67.14) |  | 755 | (29.96) |  | 36 | (1.43) |  | 11 | (0.44) |  | - | - |  | 26 | (1.03) |
| 2007 | 2282 | 1286 | (56.35) |  | 914 | (40.05) |  | 34 | (1.49) |  | 17 | (0.74) |  | - | - |  | 31 | (1.36) |
| 2008 | 1820 | 1019 | (55.99) |  | 736 | (40.44) |  | 35 | (1.92) |  | 19 | (1.04) |  | - | - |  | 11 | (0.60) |
| 2009 | 1657 | 784 | (47.31) |  | 785 | (47.37) |  | 32 | (1.93) |  | 26 | (1.57) |  | 6 | (0.36) |  | 24 | (1.45) |
| 2010 | 1811 | 555 | (30.65) |  | 1132 | (62.51) |  | 38 | (2.10) |  | 15 | (0.83) |  | 50 | (2.76) |  | 21 | (1.16) |
| 2011 | 1564 | 442 | (28.26) |  | 970 | (62.02) |  | 36 | (2.30) |  | 20 | (1.28) |  | 72 | (4.60) |  | 24 | (1.53) |
| 2012 | 1468 | 337 | (22.96) |  | 924 | (62.94) |  | 41 | (2.79) |  | 21 | (1.43) |  | 126 | (8.58) |  | 19 | (1.29) |
| 2013 | 1483 | 271 | (18.27) |  | 908 | (61.23) |  | 57 | (3.84) |  | 25 | (1.69) |  | 190 | (12.81) |  | 32 | (2.16) |
| 2014 | 1348 | 266 | (19.73) |  | 894 | (66.32) |  | 30 | (2.23) |  | 22 | (1.63) |  | 114 | (8.46) |  | 22 | (1.63) |
| 2015 | 1637 | 261 | (15.94) |  | 1173 | (71.66) |  | 38 | (2.32) |  | 14 | (0.86) |  | 131 | (8.00) |  | 20 | (1.22) |
| 2016 | 1820 | 290 | (15.93) |  | 1343 | (73.79) |  | 32 | (1.76) |  | 21 | (1.15) |  | 84 | (4.62) |  | 50 | (2.75) |
| 2017 | 1946 | 263 | (13.51) |  | 1536 | (78.93) |  | 22 | (1.13) |  | 17 | (0.87) |  | 68 | (3.49) |  | 40 | (2.06) |

Table S4.5 Age-standardized first-time offence rate for hierarchically classified illicit drugs in Taiwan, 2001-2017, stratified by age groups

(e) 50-59 years old.

| Year | Total | Heroin | |  | Others | |  |  |  |  |
| --- | --- | --- | --- | --- | --- | --- | --- | --- | --- | --- |
|  |  | N | % |  | N | % |  |  |  |  |
| 2001 | 1685 | 162 | (61.60) |  | 108 | (0.00) |  |  |  |  |
| 2002 | 2599 | 297 | (67.08) |  | 161 | (0.00) |  |  |  |  |
| 2003 | 2339 | 299 | (70.37) |  | 147 | (0.00) |  |  |  |  |
| 2004 | 2807 | 363 | (63.91) |  | 192 | (0.00) |  |  |  |  |
| 2005 | 2939 | 423 | (61.59) |  | 256 | (0.00) |  |  |  |  |
| 2006 | 2520 | 427 | (67.14) |  | 222 | (0.00) |  |  |  |  |
| 2007 | 2282 | 387 | (56.35) |  | 306 | (0.00) |  |  |  |  |
| 2008 | 1820 | 300 | (55.99) |  | 220 | (0.00) |  |  |  |  |
| 2009 | 1657 | 284 | (47.31) |  | 288 | (0.00) |  |  |  |  |
| 2010 | 1811 | 203 | (30.65) |  | 368 | (0.00) |  |  |  |  |
| 2011 | 1564 | 174 | (28.26) |  | 346 | (0.00) |  |  |  |  |
| 2012 | 1468 | 146 | (22.96) |  | 288 | (0.00) |  |  |  |  |
| 2013 | 1483 | 122 | (18.27) |  | 302 | (0.00) |  |  |  |  |
| 2014 | 1348 | 106 | (19.73) |  | 310 | (0.00) |  |  |  |  |
| 2015 | 1637 | 138 | (15.97) |  | 432 | (0.00) |  |  |  |  |
| 2016 | 1820 | 128 | (15.96) |  | 431 | (0.00) |  |  |  |  |
| 2017 | 1946 | 147 | (13.54) |  | 509 | (0.00) |  |  |  |  |
